# *Limosilactobacillus reuteri* DSM 17938 and ATCC PTA 6475 for the treatment of moderate to severe irritable bowel syndrome in adults: a randomized controlled trial

**DOI:** 10.3389/fgstr.2023.1296048

**Published:** 2024-01-04

**Authors:** Silvia Cruchet, Sandra Hirsch, Diana Villa-López, Mucio Moreno-Portillo, Juan C. Palomo, Ana T. Abreu-Abreu, Juan M. Abdo-Francis, Carlos Jiménez-Gutiérrez, Martin Rojano, Gabriel López-Velázquez, Pedro Gutiérrez-Castrellón

**Affiliations:** ^1^ Instituto de Nutrición y Tecnología de los Alimentos (INTA), Universidad de Chile, Santiago de Chile, Chile; ^2^ Gastroenterology Department, Hospital General Dr. Manuel Gea González, Mexico City, Mexico; ^3^ Gastroenterology Department, Hospital Ángeles Pedregal, Mexico City, Mexico; ^4^ Gastroenterology Department, Hospital Ángeles Acoxpa, Mexico City, Mexico; ^5^ Laboratorio de Biomoléculas y Salud Infantil, Instituto Nacional de Pediatría, Mexico City, Mexico; ^6^ Traslational Research Division, International Scientific Council for Probiotics, Mexico City, Mexico

**Keywords:** *L. reuteri* DSM 17938, *L. reuteri* ATCC PTA 6475, irritable bowel syndrome, adults, randomized controlled (clinical) trial

## Abstract

**Background:**

Irritable bowel syndrome (IBS) is a common functional gastrointestinal disorder in adults. Systematic reviews with meta-analyses have demonstrated the efficacy and safety of probiotics in improving symptoms of IBS.

**Aim:**

The aim of the study was to demonstrate the efficacy and safety of *Limosilactobacillus reuteri* (*L. reuteri*) DSM 17938 combined with *L. reuteri* ATCC PTA 6475 regarding improving the symptoms associated with IBS in adults.

**Methods:**

A randomized, double-blind, placebo-controlled clinical trial was conducted in 140 adults aged 18 years to 65 years with a diagnosis of IBS (based on the Rome IV criteria). After 2 weeks of washout, subjects were randomized to receive either 2 × 10^8^ colony-forming units (CFUs) of *L. reuteri* DSM 17938 combined with *L. reuteri* ATCC PTA 6475 plus standard of care or placebo plus standard of care for 14 weeks, followed by a post-intervention period of 2 additional weeks. Changes in gastrointestinal symptoms (as measured with the GSRS-IBS), stool pattern (as measured with the Bristol scale), quality of life, depression and anxiety, frequency of adverse events, and fecal calprotectin concentrations were evaluated.

**Results:**

In total, 70 subjects were allocated to receive *L. reuteri* and 70 were allocated to receive placebo. During the pre-randomization phase, no differences were observed between the groups in terms of IBS-associated symptoms and stool consistency. Starting at week 6 of the intervention, subjects in group *L. reuteri* showed a significant improvement in IBS-associated symptoms (*p* < 0.01). A significant improvement was also observed in fecal calprotectin concentration in the *L. reuteri* group at the end of interventions (30.2 ± 11.8 mg/g of stool in the *L. reuteri* group and 41.6 mg/g ± 10.7 mg/g in the placebo group; *p* = 0.019). The frequency of adverse events was similar between groups.

**Conclusions:**

A twice-a-day intervention for 14 weeks is safe and effective, reduces the symptoms associated with IBS in adults aged 18 years to 65 years, improves stool consistency, and reduces symptoms associated with anxiety after 6 weeks.

## Background

Irritable bowel syndrome (IBS) is the most common functional gastrointestinal disorder in adults. It is characterized by chronic abdominal pain and changes in bowel habits without an identifiable organic cause. Its prevalence is approximately 10%–15% (1–5). Annually, IBS accounts for approximately 3.1 million outpatient medical visits in the United States, with a cost exceeding $20 billion (6, 7). It is most common in women and in people aged between 20 years and 40 years (8).

IBS is characterized clinically by chronic abdominal pain of variable intensity and periodic exacerbations, changes in stool form (diarrhea, constipation, or both), and the sensation of incomplete evacuation, even when the rectum is empty, among other characteristics. It can be associated with various extraintestinal symptoms, such as altered sexual function, dysmenorrhea, dyspareunia, and increased urinary frequency and urgency (8, 9). Its pathogenesis is complex, and various intestinal motor function alterations, a prolonged gastrointestinal transit time, an exaggerated motor response, the hyperexcitability of various receptors on the intestinal wall to stimuli, such as histamine, nitric oxide, and proteases, and changes in intestinal microbiota profiles, among other factors, have been described as generators of the syndrome (9).

Its definition has changed over time. The ROME IV criteria describe four subtypes: IBS with predominant constipation (i.e., hard or lumpy stools in ≥ 25% of evacuations or soft or watery in < 25% of evacuations), IBS with predominant diarrhea (i.e., watery or soft stools in ≥ 25% of evacuations/hard or lumpy stools in < 5% of evacuations), mixed IBS (i.e., hard or lumpy stools in ≥ 25% of evacuations/watery stools in ≥ 25% of evacuations), and undetermined IBS (10–12).

From a therapeutic point of view, modifying dietary habits represents one of the most frequently used treatments (13). Increasing fiber intake represents one of the most common dietary modifications. Considering the changes observed in the intestinal microbiota profiles in these patients, the beneficial effects of fiber may reflect the increase in the production of short-chain fatty acids and their actions as prebiotics (14). Another dietary alternative is the low-FODMAPs (fermentable oligosaccharides, disaccharides, monosaccharides, and polyols) diet, which continues to generate controversies (15–24).

Considering the changes that have been observed for several years in the intestinal microbiota profiles in the subjects with IBS, the coadjuvant role of probiotics in controlling gastrointestinal symptoms was analyzed. In 2015, a systematic review was published in which 24 clinical trials were included, with 1,793 subjects. A significant improvement in abdominal pain (RR 1.96, 95% CI 1.14 to 3.36; *p* = 0.01), an improvement in the global symptoms scale score (RR 2.4, 95% CI 1.13 to 5.21; *p* = 0.02), and a specific effect on abdominal distension, pain, inflammation, and flatulence (SMD −2.57, 95% CI −13.05 to −0.92) were observed (25).

Recently, a meta-analysis on the impact of the administration of probiotics in adults with IBS was published. A total of 35 clinical trials were included in the study, with more than 3,452 subjects; the results indicated that, compared with placebo, probiotics reduced the incidence of persistent symptoms (RR 0.79, 95% CI 0.70 to 0.89; *p* 0.0001) and improved global symptoms and the abdominal pain score (SMD –0.25, 95% CI –0.36 to –0.14; *p* = 0.00001), abdominal distension score (SMD −0.15, 95% CI −0.27 to −0.03; *p* = 0.01), and flatulence score (SMD −0.20, 95% CI −0.35 to −0.05; *p* = 0.01), without conclusively establishing the predominance of any particular type of probiotic (26).

*Limosilactobacillus reuteri* DSM 17938 and *L. reuteri* ATCC PTA 6475 are two strains of *L. reuteri* species, with specific modes of action as identified *in vitro* and in animal models, showing clear anti-inflammatory potential as well as intestinal epithelial integrity and gut homeostasis capabilities. Both strains showed tolerance to gastric juice and no viability reduction after simulated gastric transit over 180 min (27). *L. reuteri* ATCC PTA 6475 showed higher adhesion capacity to epithelial cell lines and a mucus layer *in vitro* than *L. reuteri* DSM 17938 (27, 28). *L. reuteri* DSM 17938 has been shown to improve epithelial barrier function by increasing the expression of tight junction proteins and dramatically increasing enterocyte migration and proliferation in the neonatal mouse intestine (29). Interestingly, *L. reuteri* ATCC PTA 6475 has been shown to support gastrointestinal homeostasis by inducing histamine responses that suppress the development of inflammation-associated colon cancer in mice (30), while *L. reuteri* DSM 17938 showed strong antipathogenic activity through the induction of reuterin *in vitro* (31). Moreover, while *L. reuteri* ATCC PTA 6475 supports important gut–brain signaling pathways by inducing oxytocin in an autism spectrum disorder mouse model (32), *L. reuteri* DSM 17938 has been shown to reduce TRPV1 pain receptor signaling in mice (33). Given the mechanisms induced by these two *L. reuteri* strains, we decided to explore their effect in the context of IBS. The primary endpoint of this trial was to evaluate the efficacy of administering *L. reuteri* DSM 17938 and *L. reuteri* ATCC PTA 6475 for a total of 14 weeks on the global clinical improvements in gastrointestinal symptoms, evaluated using the GSRS-IBS. Second, we evaluated the effects of the intervention on the consistency of bowel movements, quality of life, depression and anxiety scores, fecal calprotectin levels, and frequency of potentially related adverse events.

## Materials and methods

This was a randomized, double-blind, multi-center clinical trial with two parallel treatment arms that was approved by the Research Committee and Research Ethics Committee of the Institute of Nutrition and Food Technology, Chile, and by the Research and Research Ethics Committees of Dr. Manuel Gea González General Hospital, Mexico (approval number 04–17–2018); the study is registered at clinicaltrials.gov as NCT04037826. A total of 140 adults aged 18 years to 65 years of either sex with a body mass index of ≤ 3 kg/m^2^ and a diagnosis of IBS based on the Rome IV criteria (i.e., recurrent abdominal pain, on average at least once per week in the last 3 months, related to defecation, associated with a change in the frequency of stools, and/or associated with a change in the appearance of bowel movements) were included; the participants were identified at the gastroenterology departments of the aforementioned institutions between 27 March 2018 and 22 August 2019, with completion of follow-up occurring in December 2019. Subjects with relevant systemic, organic, or metabolic diseases other than IBS; subjects with a recent history of previous major abdominal surgery; and subjects who had consumed antibiotics, proton pump inhibitors, H_2_ antagonists, or probiotics for at least 48 continuous hours in the 2 weeks before the baseline evaluation were excluded from the study. Considering the results published in 2016 by Yan Zhang et al. (34) in which an improvement in the global score of the IBS symptom severity scale (GSRS-IBS) was identified in 700 patients with IBS assigned to the probiotic group, compared with 575 patients with IBS included in the control group (53.3% vs. 27.7%), with an alpha error of 5%, a power of 80%, and a potential loss to follow-up of 20%, the minimum sample size was calculated as 70 subjects per treatment arm, for a total of 140 subjects in the study. Once the selection criteria were met, the subjects who signed an informed consent form were invited to participate in a 2-week pre-randomization evaluation period; the frequency and intensity of their gastrointestinal manifestations were evaluated using the gastrointestinal symptoms rating scale specific to IBS (GSRS-IBS) (35). The subjects were standardized in two sessions to use GSRS-IBS by sub-investigators. Before the recruited subjects started to fill the GSRS-IBS daily report form, they were requested to practice on a dummy version until they understand and do not have mistakes. After that they were allowed to fill the daily report form. The characteristics of their bowel movements were evaluated using the Bristol scale, the quality-of-life score was evaluated using IBS QoL Score (36), and depression and anxiety by the Goldberg Scale (37). FODMAPs and concomitant medications received during the period were also evaluated. The eating pattern was evaluated by requesting subjects to report in the diary report form 2 days during the week and 1 day during the weekend for the frequency and number of foods. Once the 2-week pre-randomization period was completed, the subjects were given an appointment for a new evaluation. For those subjects who met the IBS criteria, randomization was carried out to blindly determine who received *L. reuteri* ‘Gastrus’ (*L. reuteri* DSM 17938 and *L. reuteri* ATCC PTA 6475; BioGaia, Stockholm, Sweden), which contained a minimum of 2 × 10^8^ CFU, one chewable tablet twice daily, for a minimum total daily dose of 4 × 10^8^ CFU/day for 14 weeks, or placebo at the same frequency and for the same time interval. The placebo product was of identical composition except that it lacked bacteria. The study product was kept refrigerated (2°C–8°C) during the study period. To maintain adherence, subjects received a phone call every week to evaluate follow-up and reinforce the use of the investigational product. On every in-place visit, the investigator reinforced the importance of continuing to take the products.

The allocation of interventions was completely blinded for both the researcher and the participant. The sponsor carried out centralized randomization and the codes were kept safe by the non-blinded pharmacist until the end of the trial. Before the start of the interventions, and in addition to the clinical scales, a baseline sample of fecal matter was obtained to measure fecal calprotectin concentrations. During the 14-week intervention period, the GSRS-IBS, the Bristol scale, the quality-of-life scale, and the depression and anxiety scale were administered weekly. A second fecal sample was obtained at the end of the intervention to evaluate changes in fecal calprotectin values. Because of the high prevalence of lactose intolerance in Chile, a lactose intolerance test was made for Chilean subjects, to eliminate lactose intolerance as a cause of some of the abdominal complaints.

At the end of the intervention period and 14-week follow-up, the subject was asked to remain in the study for an additional 2 weeks, without intervention, to evaluate the potential changes in the GSRS-IBS, Bristol scale, quality-of-life, and depression and anxiety scores.

Throughout the intervention phase and for the additional 2-week surveillance phase, the feeding pattern, adherence to treatment, use of other concomitant medications, and frequency and severity of adverse events were evaluated. The subjects reported in the diary potential adverse events, and the principal investigators classified them as related or unrelated to the intervention. The diary report form (DRF) reported the type and amount of food consumed, the use of any medication during their participation in the study, and any adverse events. The journal included written instructions for filling out the forms. The information reported by subjects on the DRF was transcribed manually by investigators on a case report form (CRF) for further analysis. To ensure compliance with respect to the completion of the journal and follow-up visits, the study staff made periodic telephone calls to the study subjects to remind them of upcoming visits and to ensure that they fully understood the necessary instructions for correctly filling out the journal. All the collected information is presented using descriptive statistics, including the number of observations, missing observations, minimum value, median, maximum value, mean, and standard deviation. The *p*-values and the calculation of the 95% confidence intervals for the GSRS-IBS were performed as two-tailed tests. The changes in the GSRS-IBS and Bristol scores were analyzed using a generalized linear model for repeated measures. A one-way analysis of variance (ANOVA) was used to compare depression and anxiety scores and fecal calprotectin levels between groups. The changes in quality-of-life scores across time were analyzed using generalized linear models. For each hypothesis test, a *p*-value < 0.05 was considered significant. SPSS version 25 for Mac (IBM Corporation, Armonk, NY, USA) was used for all statistical analyses.

## Results

**Figure 1:**
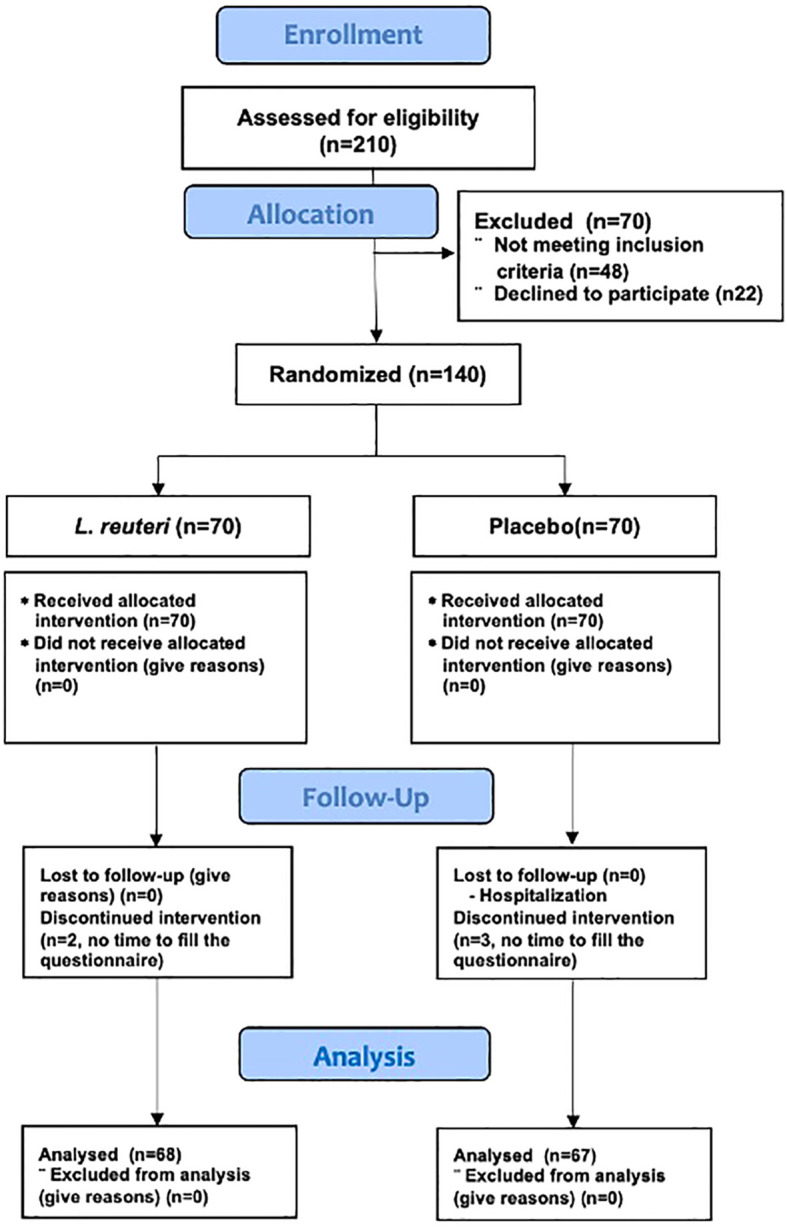
CONSORT Flow Diagram.

A total of 210 potential subjects were screened to participate in the study. A total of 140 patients were selected to be randomized to either the *L. reuteri* group (*n* = 70) or the placebo group (*n* = 70). In total, 20 subjects were recruited in Chile (10 in the *L. reuteri* group and 10 in the placebo group) and 120 in México (60 in the *L. reuteri* group and 60 in the placebo group). The causes of screening failure are included in the Consolidated Standards of Reporting Trials (CONSORT) flowchart. At the second visit, five Chilean patients withdrew because they were not willing to fill out the questionnaires or come to the control sessions due to a lack of time. (**Figure 1**: CONSORT flowchart for the study.).

Because the analysis by subgroups (Chilean cohort vs. Mexican cohort) did not show any significant differences (see **Supplementary Material**), we present the main results as a global cohort, comparing 68 subjects allocated to the *L. reuteri* group and 67 to the control group.

The age of the subjects was 39.4 years ± 9.98 years in the *L. reuteri* group and 40.2 years ± 11.1 years in the placebo group (*p* = NS). Most of the recruited subjects were female (82% in the *L. reuteri* group and 78% in the placebo group; *p* = NS). Ingestion of FODMAPs before recruitment was reported as low, at 85% and 80% in the *L. reuteri* and placebo groups, respectively (*p* = NS). Except for greater smoking, a greater frequency of physical activity, and higher levels of calprotectin in the *L. reuteri* group at admission, no significant differences were found (**Table 1**).

**Table 1:** Base information at recruitment.

Parameter	*L. reuteri* group (*n* = 70)	Placebo group (*n* = 70)
Age (years) (x ± s.d.) [Min.–max.]	39.4 ± 9.98	40.2 ± 11.1
Sex, female (%)	82	78
Scholarship (years) (x ± s.d.) [Min.–max.]	19 ± 4	18 ± 4
Blood group (%) O positive O negative A positive A negative B positive B negative AB positive AB negative	67 5 18 2 7 1 0 0	73 2 12 5 5 2 1 0
Smoking status (%) Duration (years) (x ± s.d.) [Min.–max.] Frequency (cigarettes/day) (x ± s.d.) [Min.–max.]	25* 5.8 ± 9.0 3.0 ± 2.7	8 3.9 ± 6.0 2.6 ± 3.4
Alcohol ingestion (%) Duration (years) (x ± s.d.) [Min.–max.] Drinks/week (x ± s.d.) [Min.–max.]	15 3.1 ± 2.9 2.5 ± 1.0	13 3.4 ± 3.3 3.3 ± 1.4
Physical activity (%) Frequency/week (x ± s.d.) [Min.–max.] Mild intensity (%) Moderate intensity (%)	53* 3.6 ± 1.9 81.3 90.5	35 3.8 ± 1.5 18.7 9.5
FODMAP ingestion Low (%) Moderate (%)	85 15	80 20
Weight (kg) (x ± s.d.) [Min.–max.]	69.2 ± 10.2	70.0 ± 11.1
Height (m) (x ± s.d.) [Min.–max.]	1.60 ± 0.07	1.61 ± 0.08
Body mass index (kg/m^2^)	26.9 ± 3.4	30.1 ± 2.7
Drug consumption (%) NSAIDs Antiepileptics Laxatives Contraceptives Anorexigenics Vitamins Antidepressives	3.3 1.7 1.7 0.0 1.7 1.7 1.7	5.0 0.0 1.7 1.7 0.0 0.0 0.0
Fecal calprotectin concentration (mg/g feces)	64.8 ± 17.8*	56.2 ± 14.2

*p<0.05, **p<0.01, otherwise NS. FODMAP, Fermentable oligosaccharides, disaccharides, monosaccharides and polyols; NSAIDs, Non Steroidals Antiinflammatory Drugs.

**Figure 2:**
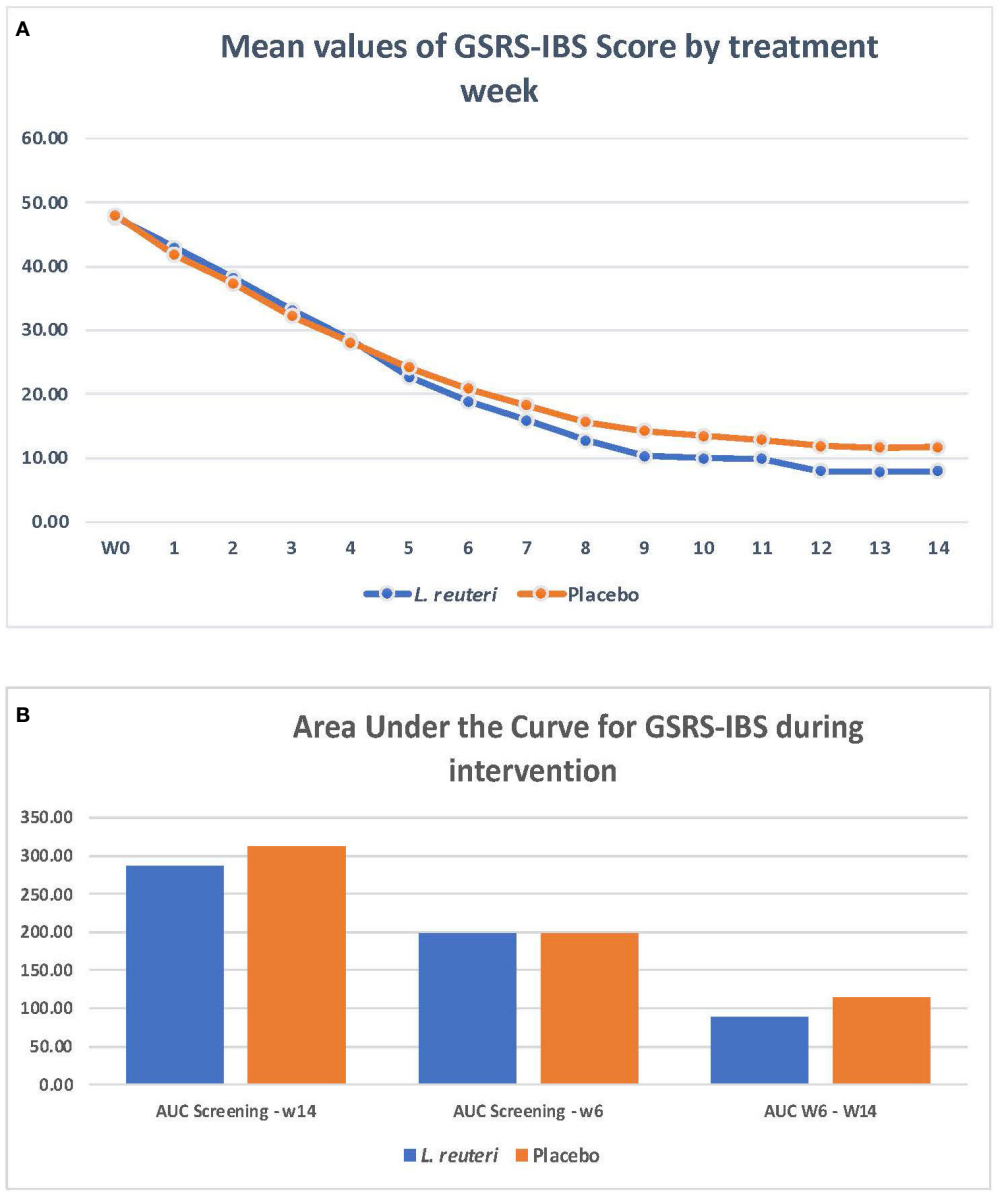
*Limosilactobacillus reuteri* DSM 17938 + ATCC PTA 6475 and improvement on irritable bowel syndrome symptoms.

During the pre-randomization phase, no differences were observed in the GSRS-IBS (47.8 ± 8.4 in the *L. reuteri* group and 48.3 ± 9.8 in the placebo group; *p* = 0.74), Bristol scale (3.5 ± 1.17 in the *L. reuteri* group and 3.7 ± 0.97 in the placebo group; *p* = 0.24), quality-of-life (100 ± 18.7 in the *L. reuteri* group and 99 ± 17.8 in the placebo group), anxiety (5.9 ± 2.81 in the *L. reuteri* group and 6.6 ± 2.34 in the placebo group), or depression scores (2.8 ± 1.83 in the *L. reuteri* group and 2.9 ± 2.13 in the placebo group) (see appendix for **Supplementary Material Tables S1, 2**). During the intervention phase, the adjusted model showed a clear improvement in the severity of IBS-related symptoms observed in the *L. reuteri* group, starting from week 6 (18.86 ± 5.47 vs. 20.93 ± 5.55, 95% CI −3.91 to −0.23; *p* < 0.05) and maintained to the end of the 14-week intervention (7.94 ± 6.96 vs. 11.71 ± 6.54, 95% CI −6.03 to −1.51; *p* < 0.001) (**Figure 2A**, **Supplementary Table S3**). To handle the risk of mass significance for multiple measurements, we measured the area under the curve (AUC) for the GSRS-IBS score, as calculated by the means of the trapezoid formula and by three time periods, and showed that there was a statistically significant difference between the groups from the screening of participants, which was more pronounced from week 6 of the intervention onward (**Figure 2B**, **Supplementary Table S4**).

**Figure 3:**
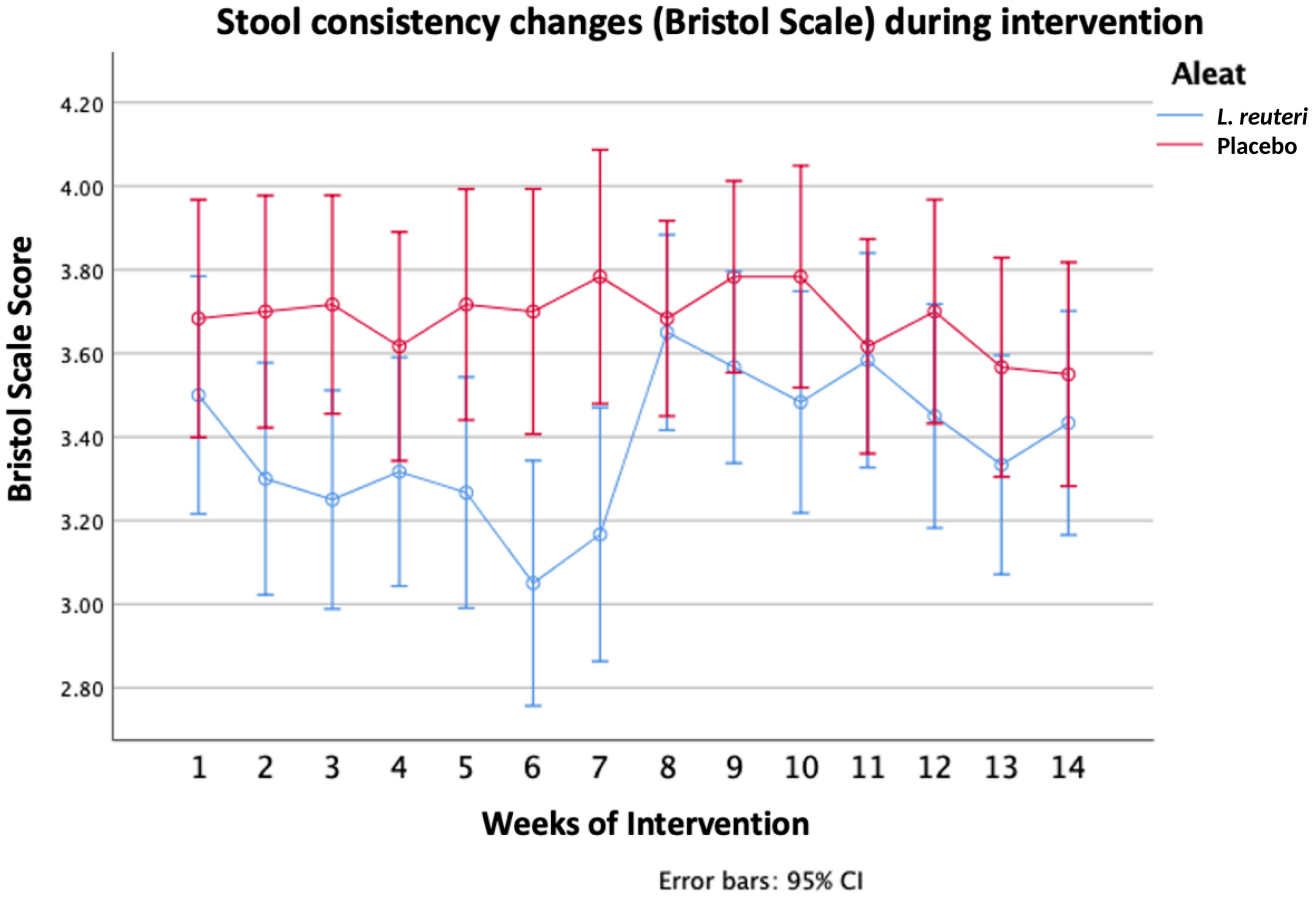
Stool consistency changes (Bristol Scale) during intervention.

The main improvement of IBS symptoms was related to abdominal pain, pain relieved by a bowel action, bloating, passing gas, and visible distension, which was reduced and was significantly better in the *L. reuteri* group than in the placebo group (**Table 2**) Stool consistency, as evaluated by the Bristol scale, did not show significant differences between groups. (**Figure 3**) As an exploratory analysis, we divided the samples by the type of IBS, identifying 23 constipated types, 33 predominantly diarrheic types, and 84 mixed types. Except for a significant improvement on the Bristol scale for diarrheic types in the first 8 weeks in favor of *L. reuteri*, we did not observe significant differences that could have been produced by the small sample size of the subgroups (**Supplementary Figures 4, 5**).

**Table 2:** Evolution of IBS symptoms during the intervention (as based on GSRS-IBS score).

Parameter	Weeks of intervention			
	1 (x ± s.d.)	5 (x ± s.d.)	10 (x ± s.d.)	14 (x ± s.d.)
Abdominal pain	A: 4.0 ± 1.2 B: 4.1 ± 1.3	A: 1.1 ± 1.0^a,b^ B: 2.0 ± 1.1	A: 1.0 ± 0.2^a,b^ B: 1.8 ± 0.5	A: 1.0 ± 0.3^a,b^ B: 1.6 ± 0.4
Pain relieved by a bowel action	A: 4.0 ± 1.2 B: 4.2 ± 1.3	A: 1.3 ± 0.9^a,b^ B: 2.2 ± 0.7	A: 1.1 ± 0.4^a,b^ B: 1.7 ± 0.4	A: 1.1 ± 0.5^a,b^ B: 1.9 ± 0.6
Bloating	A: 4.4 ± 1.2 B: 4.6 ± 1.3	A: 2.0 ± 0.6^a,b^ B: 2.9 ± 0.8	A: 1.2 ± 0.5^a,b^ B: 2.1 ± 0.4	A: 1.2 ± 0.3^a,b^ B: 1.8 ± 0.4
Passing gas	A: 4.2 ± 1.2 B: 4.2 ± 1.5	A: 3.2 ± 0.9 B: 3.3 ± 0.7	A: 1.0 ± 0.6^a,b^ B: 1.9 ± 0.3	A: 1.3 ± 0.4^a,b^ B: 2.0 ± 0.5
Visible distension	A: 4.2 ± 1.7 B: 3.8 ± 1.8	A: 1.4 ± 1.0^a,b^ B: 2.6 ± 0.9	A: 1.0 ± 0.4^a,b^ B: 2.0 ± 0.7	A: 1.0 ± 0.3^a,b^ B: 1.6 ± 0.6
Constipation	A:2.9 ± 1.7 B: 3.1 ± 1.8	A:3.0 ± 1.1 B: 3.3 ± 0.8	A:1.4 ± 0.8 B: 1.3 ± 0.4	A:1.1 ± 0.6 B: 1.0 ± 10.3
Hard stools	A: 3.1 ± 1.6 B:3.2 ± 1.6	A: 3.1 ± 1.1 B:3.5 ± 0.7	A: 1.3 ± 0.5 B:1.3 ± 0.3	A: 1.0 ± 0.5 B:1.1 ± 0.4
Diarrhea	A: 4.0 ± 1.5 B: 3.6 ± 1.6	A: 2.3 ± 1.3 B: 2.7 ± 0.7	A: 1.2 ± 0.3 B: 1.4 ± 0.4	A: 1.0 ± 0.6 B: 1.2 ± 0.4
Loose stools	A: 3.5 ± 1.8 B: 3.4 ± 1.6	A: 2.1 ± 1.0 B: 1.9 ± 0.9	A: 1.0 ± 0.6 B: 1.2 ± 0.8	A: 1.0 ± 0.3 B: 1.3 ± 0.6
Urgent need for bowel movement	A: 4.2 ± 1.5 B: 4.2 ± 1.7	A: 2.2 ± 0.7 B: 2.4 ± 1.1	A: 1.3 ± 0.4 B: 1.4 ± 0.9	A: 1.1 ± 0.6 B: 1.3 ± 0.7
Incomplete bowel emptying	A: 3.7 ± 1.6 B: 4.0 ± 1.7	A: 2.0 ± 1.4 B: 2.5 ± 0.9	A: 1.1 ± 0.7 B: 1.8 ± 0.6	A: 1.0 ± 0.4 B: 1.1 ± 0.4
Fullness shortly after meal	A: 3.7 ± 1.8 B: 3.9 ± 1.9	A: 1.4 ± 1.2 B: 1.7 ± 0.7	A: 1.2 ± 0.4 B: 1.6 ± 0.5	A: 1.0 ± 0.5 B: 1.3 ± 0.5
Fullness long after eating	A: 3.3 ± 1.1 B: 3.1 ± 1.3	A: 2.0 ± 0.8 B: 1.1 ± 0.6	A: 1.1 ± 0.3 B: 1.6 ± 0.4	A: 1.2 ± 0.4 B: 1.1 ± 0.2

Kruskal–Wallis test.

^a^significant differences within groups; ^b^significant differences between groups; A, *L. reuteri* group; B, placebo group.

A significant improvement was observed at the end of the 14 weeks of treatment in the *L. reuteri* group regarding the quality-of-life score and anxiety levels, with a trend toward improving anxiety (**Table 3**).

**Table 3:** Quality of life, anxiety, and depression scores in the treatment phase (14 weeks).

Parameter (x ± s.d.)	*L. reuteri* group (*n* = 68)	Placebo group (*n* = 67)
Week 7 of intervention QoL-IBS score	124.0 ± 16.32*	103.1 ± 11.1
Goldberg-Anxiety	1.9 ± 0.81*	3.7 ± 2.75
Goldberg-Depression	0.8 ± 0.63	1.5 ± 1.65
Week 14 of intervention QoL-IBS Score	131.6 ± 15.38*	108.4 ± 7.2
Goldberg-Anxiety	1.1 ± 0.92*	2.4 ± 3.12
Goldberg-Depression	0.9 ± 0.2	1.2 ± 1.86

*p<0.05, **p<0.01, otherwise NS. QoL-IBS, Quality of Life Irritable Bowel Syndrome Score.

Fecal calprotectin values in the *L. reuteri* group were significantly lower at the end of 14 weeks of treatment than in those in the placebo group (30.2 mg/g ± 11.8 mg/g of stool vs. 41.6 mg/g ± 10.7 mg/g of stool; *p* = 0.019). Regarding the safety of the interventions, no significant differences were observed in the frequency of adverse events reported in the first 30 min after ingesting products under research during the intervention period (**Table 4**). Data related to lactose intolerance did not modify the effect of the intervention, so we do not present it here.

**Table 4:** Frequency of adverse events during the treatment phase (14 weeks).

Adverse events (%)	*L. reuteri* group (*n* = 70)	Placebo group (*n* = 70)
Regurgitation	20	19
Abdominal pain	2	3
Passing gas	32	29
Reduction stool consistency	6	4

*p<0.05, **p<0.01, otherwise NS.

At the end of the intervention period, the subjects were under surveillance for 2 additional weeks to evaluate the evolution of IBS symptoms and changes on the Bristol, quality-of-life, anxiety, and depression scale scores. The changes observed during the intervention phase were maintained during this post-treatment period (**Table 5**).

**Table 5:** GSRS-IBS evaluation (post-treatment observational phase).

Parameter (x ± s.d.)	*L. reuteri* group (*n* = 68)	Placebo group (*n* = 67)	*p*-value
Abdominal pain	1 ± 0.8	3 ± 0.4	0.01
Pain relieved by a bowel action	1 ± 0.9	2 ± 1.1	0.03
Bloating	1 ± 1.1	2 ± 0.4	0.02
Passing gas	1 ± 0.8	2 ± 0.7	0.02
Visible distension	1 ± 1.0	3 ± 0.6	0.01

## Discussion and conclusions

IBS represents one of the most common brain–gut–microbiome interaction disorders, with significant symptoms and a significant deterioration in quality of life; it is therefore associated with a significant economic impact due to frequent episodes of symptomatology and, thus, increased work absenteeism. Although its exact causes are unknown, altered visceral hypersensitivity (38, 39), chronic immune activation with the development of low-grade inflammation (40–43), and changes in the alpha and beta diversity of the intestinal microbiome have been described (44–47).

In this sense, probiotics have shown different impacts through modulating hypersensitivity, reducing low-grade inflammation, and modifying alpha or beta diversity in the intestinal microbiome (48–57). The different strains of *Lactobacillus* have been shown to reduce pro-inflammatory tumor necrosis factor alpha (TNF-α) production in peripheral blood mononuclear cells in healthy subjects. A combination of *Lactobacillus* strains has been shown to reduce pro-inflammatory cytokines in the digestive tract of subjects with neuroinflammation (48–50).

Some *Bifidobacteria* have been reported to enhance the immune responsivity of mucosal surfaces and have shown promising benefits for healthy adults (51). Other strains of *Bifidobacterium* also decrease lipopolysaccharide (LPS) concentrations, which may reduce inflammatory cytokine production (52) and reduce cytokine- and T-cell-mediated inflammation (53). These effects are likely to improve the intestinal barrier and immune function and may also have a role in alleviating functional gastrointestinal disorders such as IBS (54).

*Lactobacillus* strains modulate visceral hypersensation and alleviate visceral pain in some animal models, increasing enterocyte opioid and cannabinoid receptor expression and reducing the activity of sodium channels (55, 56). Studies on healthy mice with modified microbiota secondary to the use of antibiotics showed inhibition of VH associated with inflammation after administration of *Lactobacillus*, a clear anti-inflammatory response, and inhibition of afferent pain pathways (57).

Concerning strain specificity and dose-specific effects of probiotics on IBS, although in 2018, a certain differential effect was demonstrated in terms of the potency of *Lactobacillus* species and *Bifidobacteria* with respect to improving IBS symptoms (58), the reality is that, to date, it has not been possible to demonstrate the significant effect of a particular strain at a specific dose and with a determined frequency. For example, in 2018, an analysis of the efficacy of VSL3 in subjects with IBS did not find sufficient evidence to demonstrate significant efficacy regarding controlling symptoms in subjects with IBS (59).

Protecting intestinal epithelial integrity in IBS patients is crucial to avoid mucosal damage and increased permeability. We combined two *L. reuteri* probiotic strains with specific and complementary effects at the intestinal mucosa, previously established *in vitro* and *in vivo*. *L. reuteri* ATCC PTA 6475 has been shown to bind to intestinal mucus *in vitro* as well as to normalize tight junction protein expression in IPEC-2 monolayers infected by enterotoxigenic *Escherichia coli* (ETEC) (60). *L. reuteri* DSM 17938 is a well-established probiotic strain that has been shown to stimulate enterocyte migration and microbial diversity in the neonatal mouse intestine (29). Moreover, *L. reuteri* ATCC PTA 6475 and *L. reuteri* DSM 17938 have been shown to significantly reduce ETEC-induced IL-6 and TNF-α secretion in IPEC-2 cells, providing evidence for a potential mucosal anti-inflammatory effect in animals and humans (60). The scientific rationale for combining these two strains was to identify the potential synergistic effects of the combination.

In this study, we demonstrated that the use of *L. reuteri* DSM 17938 and *L. reuteri* ATCC PTA 6475 significantly reduces the clinical symptoms associated with IBS when evaluated using the GSRS-IBS score. Subjects treated with the combination of probiotics showed an 80% vs. 43% reduction in the frequency and severity of symptoms starting from week 6 of the intervention. Even when we calculated the 95% CIs for the differences between the *L. reuteri* and the placebo interventions, we identified these significant differences, which supports the significance of the effect. Improvements in symptoms were predominantly observed in abdominal pain, pain relieved by a bowel action, bloating and visible distension (which started to improve on week 5 of treatment), and passing gas improvement (starting on week 10). This effect is aligned with previous evidence for *L. reuteri* in subjects with *Helicobacter pylori* infections or functional abdominal pain, in which abdominal pain and bloating reduction were significantly reduced (61–65). This is consistent with the evidence previously cited in animal models in which these two strains modulated low-grade inflammation, increased tight junction proteins, and reduced visceral hypersensitivity. One interesting result regarding the efficacy observed in the cohort of subjects treated with probiotics is that this effect remained during the 2-week observation period after the treatment was stopped. The impact of intervention on the immune and anti-inflammatory effects of the use of *L. reuteri* had been previously observed by our group in children treated with *L. reuteri* DSM 17938, in which the effects persisted 12 weeks after intervention (66).

The quality-of-life and anxiety scores also significantly improved in subjects treated with *L. reuteri*. A significant reduction in the frequency and severity of IBS-related symptoms, mainly abdominal pain, bloating, and distension, seems to be the more logical explanation for the improvement of these two parameters. Recently, a systematic review with meta-analysis has shown that probiotics significantly reduce anxiety in adults, as evaluated by the State–Trait Anxiety Inventory (STAI) (MD, −6.88, 95% CI −12.35 to −1.41; *p* = 0.01; *I* ^2 = ^24%) (67). A growing body of evidence suggests that probiotics can modulate gut–brain interactions by regulating neurotransmitters and proteins critical for the neural excitatory–inhibitory balance, mood, cognitive functions, and memory processes (68–70). Although the pathways involved are not completely understood, they might be associated with the brain tissue’s susceptibility to changes in the inflammatory process and oxidative stress status (71, 72).

Finally, in terms of safety, potential adverse events evaluated in the first 30 min after subjects received the intervention did not show significant differences between the probiotics group and the placebo group, which is aligned with previous evidence that shows the safety and tolerability of these two strains of *L. reuteri* in toddlers, children, and adults.

The main strengths of our study are, first, the size of the sample; second, the strategy used to establish an objective monitoring period and apply validated scales over a period of 2 weeks to ensure compliance with the Rome IV criteria; third, double-blinding with an extended follow-up period of 14 weeks to identify the onset of effects and evaluate the continuity of effects across time; and, finally, the establishment of a follow-up period of 2 weeks, without intervention, to evaluate residual anti-inflammatory effects.

Among the main limitations is the inability to perform, given the sample size, a stratified analysis based on the type of IBS to evaluate a differential impact (perhaps with a greater effect among individuals with predominant constipation). The second major limitation is the lack of an analysis of the patients’ baseline and final microbiota profile to establish a correlation between the clinical changes observed and potential changes in the alpha and beta diversity of the populations of fecal microbiota.

The evidence identified in the present study, the first of its kind to demonstrate the efficacy of this combination of *L. reuteri* strains, indicates that the strains are safe and effective adjuvants for the management of gastrointestinal symptoms in adults with IBS and improve the quality of life and associated symptoms of anxiety in these patients.

This line of research should be continued to provide additional support to the results obtained herein. If possible, the interventions should be randomized within each IBS subtype, and changes in the microbiota profile and their association with pro-/anti-inflammatory biomarkers should be evaluated in at least one subsample of subjects in each stratum.

## Data availability statement

The raw data supporting the conclusions of this article will be made available by the authors, without undue reservation.

## Ethics statement

The studies involving humans were approved by Comite de Investigación y Comite de Ética de la Investigación. Hospital General Dr Manuel Gea González. The studies were conducted in accordance with the local legislation and institutional requirements. The participants provided their written informed consent to participate in this study.

## Author contributions

SC: Supervision, Writing – review & editing, Project administration. SH: Methodology, Validation, Writing – review & editing, Project administration. DV-L: Writing – review & editing, Investigation. JP: Investigation, Writing – review & editing. MM-P: Supervision, Writing – review & editing. AA-A: Writing – review & editing, Investigation. JA-F: Investigation, Writing – review & editing. CJ-G: Formal Analysis, Methodology, Writing – review & editing. MR: Conceptualization, Investigation, Writing – review & editing. GL-V: Formal Analysis, Investigation, Writing – review & editing. PG-C: Conceptualization, Methodology, Project administration, Validation, Writing – review & editing.
