# Supplementary material for: Limosilactobacillus reuteri DSM 17938 and ATCC PTA 6475 for the treatment of moderate to severe irritable bowel syndrome in adults: a randomized controlled trial
Source: Front Gastroenterol (Lausanne). 2024 Jan 4;2:1296048. doi: 10.3389/fgstr.2023.1296048 (PMC12952303; doi:10.3389/fgstr.2023.1296048)

Fig. 1 Effect of *L. reuteri* on Stool Consistency on Diarrheic IBS Subjects

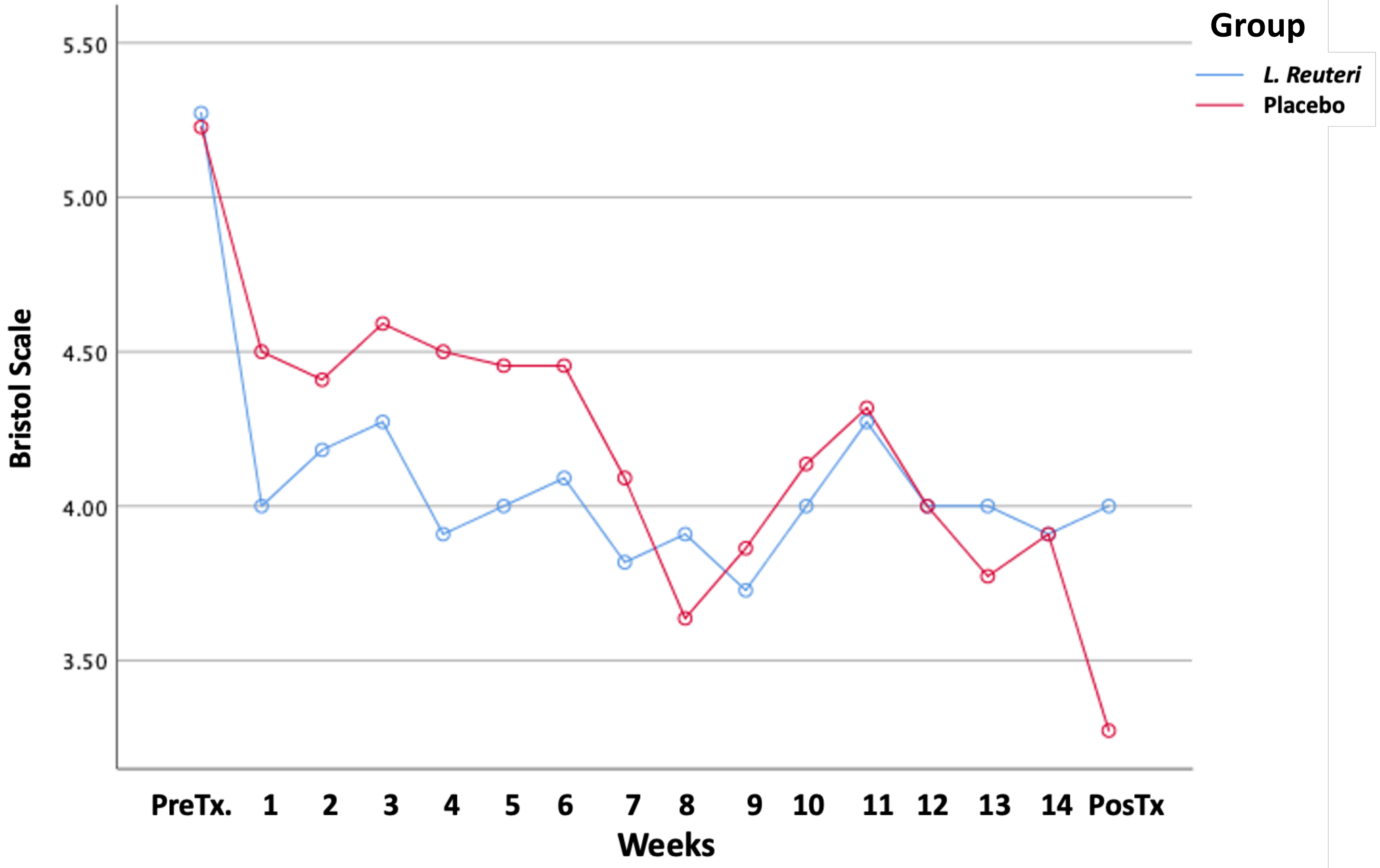

Fig. 2 Effect of L. reuteri on Stool Consistency on Constipated IBS Subjects

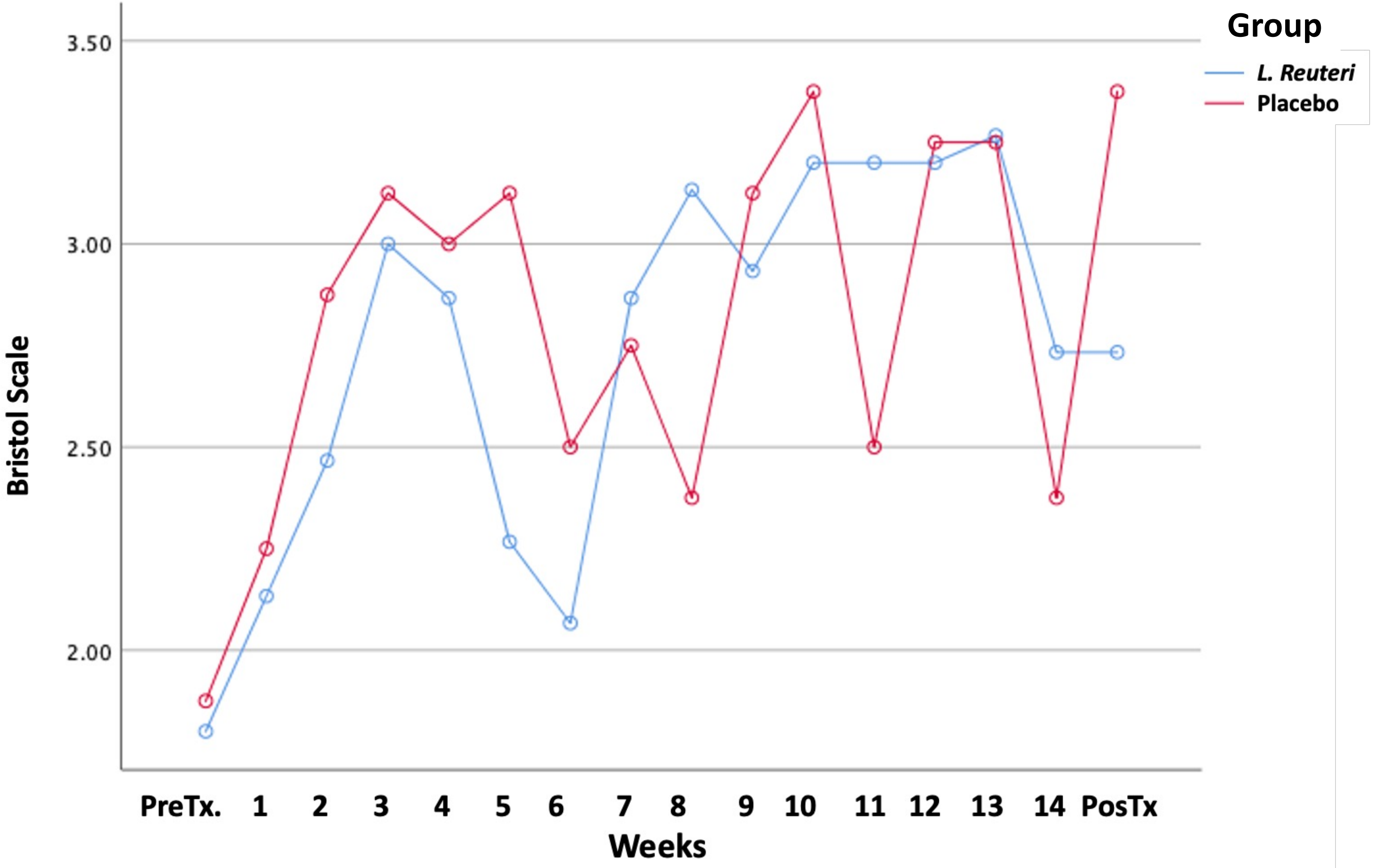

Fig. 3 Effect of L. reuteri on Stool Consistency on Mixed Type IBS Subjects

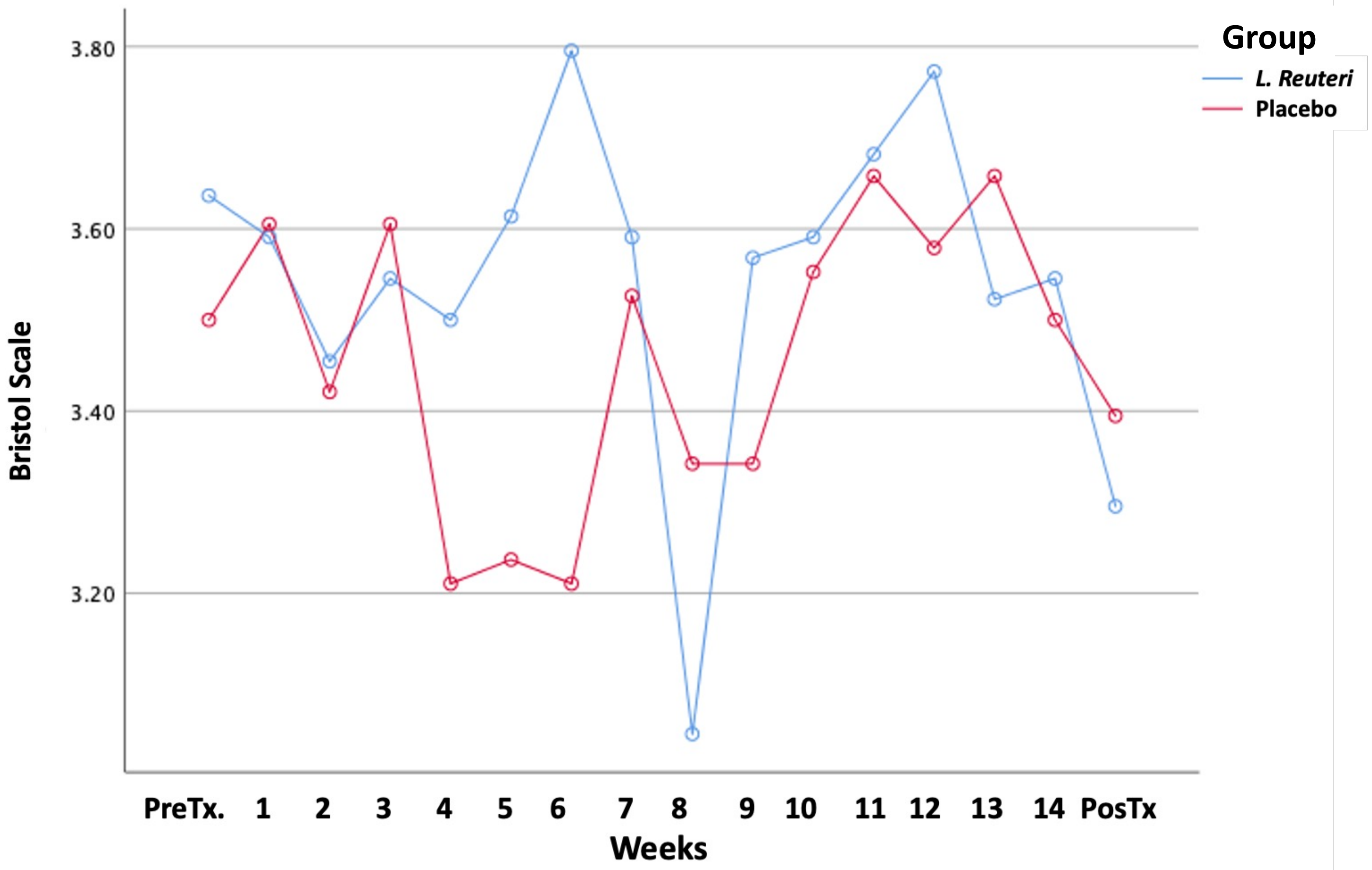

Supplement: Supplementary file 1 [file Image_1.pdf]
