# Supplementary material for: Limosilactobacillus reuteri DSM 17938 and ATCC PTA 6475 for the treatment of moderate to severe irritable bowel syndrome in adults: a randomized controlled trial
Source: Front Gastroenterol (Lausanne). 2024 Jan 4;2:1296048. doi: 10.3389/fgstr.2023.1296048 (PMC12952303; doi:10.3389/fgstr.2023.1296048)
Supplement: Supplementary file 2 [file Table_1.docx]

**Appendix. Supplementary Material**

**Table SI. GSRS-IBS Evaluation (Pre-randomization Phase)**

| **Parameter (x±s.d.)** | **Group L. reuteri**  **(n=70)** | **Group Placebo**  **(n=70)** | **p** |
| --- | --- | --- | --- |
| Abdominal pain | 5±1.2 | 4±1.3 | 0.77 |
| Pain relieved by a bowel action | 4±1.2 | 4±1.2 | 0.42 |
| Bloating | 4±0.9 | 4±0.7 | 0.40 |
| Passing gas | 6±0.8 | 5±1.2 | 0.95 |
| Visible distention | 4±1.7 | 5±1.8 | 0.31 |
| Constipation | 4±1.7 | 3±1.6 | 0.68 |
| Hard stools | 3±1.6 | 3±1.6 | 0.70 |
| Diarrhoea | 2±1.5 | 2±1.6 | 0.15 |
| Loose stools | 1±1.2 | 2±1.4 | 0.63 |
| Urgent need for bowel movement | 4±1.5 | 4±1.7 | 0.82 |
| Incomplete bowel emptying | 4±1.6 | 4±1.7 | 0.23 |
| Fullness shortly after meal | 4±1.8 | 3±1.9 | 0.62 |
| Fullness long after eating | 3±1.1 | 3±1.3 | 0.37 |

**Table SII. Bristol Scale (Pre-randomization Phase)**

| **Day (x±s.d.)** | **Group L. reuteri**  **(n=70)** | **Group Placebo**  **(**  **n=70)** | **p** |
| --- | --- | --- | --- |
| **1** | 3.2±1.3 | 3.7±1.4 | 0.07 |
| **2** | 3.6±1.5 | 3.9±1.4 | 0.27 |
| **3** | 3.4±1.6 | 4.0±1.3 | 0.06 |
| **4** | 3.7±1.7 | 3.8±1.5 | 0.77 |
| **5** | 3.5±1.6 | 3.7±1.4 | 0.47 |
| **6** | 3.5±1.5 | 3.7±1.4 | 0.40 |
| **7** | 3.6±1.6 | 3.9±1.4 | 0.27 |
| **8** | 3.6±1.6 | 3.7±1.6 | 0.86 |
| **9** | 3.5±1.7 | 3.8±1.5 | 0.22 |
| **10** | 3.5±1.5 | 4.0±1.5 | 0.07 |
| **11** | 3.7±1.6 | 3.7±1.3 | 0.95 |
| **12** | 3.6±1.5 | 3.5±1.4 | 0.95 |
| **13** | 3.5±1.6 | 3.6±1.6 | 0.69 |
| **14** | 3.3±1.5 | 3.5±1.4 | 0.50 |

**Table SIII. Min, median and max values of GSRS-IBS scores during intervention**

| Group | Statistica | Screening | Week 1 | Week 2 | Week 3 | Week 4 | Week 5 | Week 6 |
| --- | --- | --- | --- | --- | --- | --- | --- | --- |
| L. reuteri | N | 70 | 70 | 68 | 68 | 68 | 68 | 68 |
|  | Nmiss | 0 | 0 | 0 | 0 | 0 | 0 | 0 |
|  | Min | 23 | 19 | 20 | 18 | 14 | 11 | 10 |
|  | Median | 48 | 44 | 40 | 32 | 30 | 23 | 19 |
|  | Max | 80 | 69 | 57 | 51 | 43 | 34 | 34 |
|  | Mean | 47.68 | 43.00 | 38.29 | 33.23 | 28.44 | 22.69 | 18.86 |
|  | Std | 11.49 | 8.89 | 9.13 | 7.72 | 6.68 | 5.29 | 5.47 |
|  | P-value^[[1]](#footnote-1)^ | NA | NA | NA | NA | NA | NA | NA |
| Placebo | N | 70 | 70 | 67 | 67 | 67 | 67 | 67 |
|  | Nmiss | 0 | 0 | 0 | 0 | 0 | 0 | 0 |
|  | Min | 23 | 23 | 20 | 17 | 15 | 11 | 11 |
|  | Median | 48 | 41 | 38 | 33 | 29 | 24 | 21 |
|  | Max | 74 | 60 | 55 | 45 | 43 | 40 | 34 |
|  | Mean | 48.03 | 41.79 | 37.37 | 32.20 | 28.17 | 24.19 | 20.93 |
|  | Std | 11.19 | 8.56 | 8.18 | 6.52 | 6.03 | 5.82 | 5.55 |
| L. reuteri-Placebo | CI_95%_ | -4.14,3.45 | -1.70,4.13 | -1.98,3.81 | -1.36,3.42 | -1.86,2.40 | -3.36,0.36 | -3.91,-0.23 |
|  | P-value^[[2]](#footnote-2)^ | NA | NA | NA | NA | NA | NA | NA |
| P-value^[[3]](#footnote-3)^ |  | 0.7402 | 0.3182 | 0.3190 | 0.5803 | 0.6704 | 0.1050 | **0.0298** |

**Table SIII. Min, median and max values of GSRS-IBS scores during intervention (Cont…)**

| Group | Statistica | Week 7 | Week 8 | Week 9 | Week 10 |
| --- | --- | --- | --- | --- | --- |
| L. reuteri | N | 68 | 68 | 68 | 68 |
|  | Nmiss | 0 | 0 | 0 | 0 |
|  | Min | 9 | 3 | 1 | 2 |
|  | Median | 14 | 11 | 10 | 9 |
|  | Max | 26 | 23 | 21 | 22 |
|  | Mean | 15.87 | 12.81 | 10.31 | 9.94 |
|  | Std | 4.78 | 5.59 | 6.06 | 6.24 |
|  | P-value^[[4]](#footnote-4)^ | NA | NA | NA | NA |
| Placebo | N | 67 | 67 | 67 | 67 |
|  | Nmiss | 0 | 0 | 0 | 0 |
|  | Min | 8 | 3 | 2 | 2 |
|  | Median | 19 | 16 | 15 | 14 |
|  | Max | 33 | 33 | 28 | 29 |
|  | Mean | 18.27 | 15.64 | 14.23 | 13.50 |
|  | Std | 5.40 | 5.90 | 6.28 | 6.27 |
| L. reuteri-Placebo | CI_95%_ | -4.10,-0.70 | -4.75,-0.91 | -5.98,-1.85 | -5.65,-1.47 |
|  | P-value^[[5]](#footnote-5)^ | NA | NA | NA | NA |
| P-value^[[6]](#footnote-6)^ |  | **0.0124** | **0.0052** | **0.0003** | **0.0014** |

**Table SIII. Min, median and max values of GSRS-IBS scores during intervention (Cont…)**

| Group | Statistica | Week 11 | Week 12 | Week 13 | Week 14 |
| --- | --- | --- | --- | --- | --- |
| L. reuteri | N | 68 | 68 | 68 | 68 |
|  | Nmiss | 0 | 0 | 0 | 0 |
|  | Min | 0 | 0 | 0 | 0 |
|  | Median | 6 | 6 | 5 | 5 |
|  | Max | 74 | 21 | 23 | 22 |
|  | Mean | 9.93 | 7.96 | 7.84 | 7.94 |
|  | Std | 10.21 | 5.65 | 6.21 | 6.96 |
|  | P-value^[[7]](#footnote-7)^ | NA | NA | NA | NA |
| Placebo | N | 67 | 67 | 67 | 67 |
|  | Nmiss | 0 | 0 | 0 | 0 |
|  | Min | 1 | 0 | 1 | 0 |
|  | Median | 13 | 12 | 13 | 13 |
|  | Max | 29 | 28 | 23 | 24 |
|  | Mean | 12.83 | 11.89 | 11.64 | 11.71 |
|  | Std | 6.81 | 6.55 | 6.35 | 6.54 |
| L. reuteri-Placebo | CI_95%_ | -5.80,0.00 | -5.97,-1.88 | -5.90,-1.70 | -6.03,-1.51 |
|  | P-value^[[8]](#footnote-8)^ | NA | NA | NA | NA |
| P-value^[[9]](#footnote-9)^ |  | **0.0024** | **0.0007** | **0.0008** | **0.0033** |

**Table SIV. Area Under the Curve of GSRS-IBS scores during intervention**

| Statistica | AUC Screening – W14 | AUC Screening – W6 | AUC W6 – W14 |
| --- | --- | --- | --- |
| N | 70 | 70 | 68 |
| Nmiss | 0 | 0 | 0 |
| Min | 174 | 126 | 36 |
| Median | 282 | 200 | 66 |
| Max | 429 | 288 | 177 |
| Mean | 286.99 | 198.91 | 88.07 |
| Std | 60.16 | 37.53 | 43.26 |
| P-value^[[10]](#footnote-10)^ | NA | NA | NA |
| N | 70 | 70 | 67 |
| Nmiss | 0 | 0 | 0 |
| Min | 177 | 113 | 27 |
| Median | 320 | 197 | 124 |
| Max | 460 | 276 | 219 |
| Mean | 312.51 | 198.19 | 114.32 |
| Std | 61.72 | 35.26 | 43.69 |
| P-value^[[11]](#footnote-11)^ | NA | NA | NA |
|  | **0.0145** | 0.8184 | **0.0010** |

**Table SV. GSRS-IBS Evaluation (Post Treatment Phase)**

| **Parameter (x±s.d.)** | **Group A**  **(n=68)** | **Group B**  **(n=67)** | **p** |
| --- | --- | --- | --- |
| Abdominal pain | 1.1±0.3 | 2.1±0.4 | 0.01 |
| Pain relieved by a bowel action | 1.2±0.7 | 1.9±0.6 | 0.03 |
| Bloating | 1.3±1.0 | 2.1±0.3 | 0.02 |
| Passing gas | 1.0±0.4 | 1.9±0.4 | 0.02 |
| Visible distention | 1.0±0.1 | 2.0±0.4 | 0.01 |
| Constipation | 1.1±0.6 | 1.3±0.3 | 0.06 |
| Hard stools | 1.0±0.9 | 2.0±0.9 | 0.03 |
| Diarrhoea | 1.2±0.4 | 1.3±0.4 | 0.67 |
| Loose stools | 1.1±0.7 | 1.2±0.6 | 0.18 |
| Urgent need for bowel movement | 1.0±0.3 | 2.2±0.6 | 0.01 |
| Incomplete bowel emptying | 1.2±0.9 | 2.0±0.8 | 0.03 |
| Fullness shortly after meal | 1.1±0.7 | 2.2±0.7 | 0.02 |
| Fullness long after eating | 1.0±0.6 | 1.3±0.6 | 0.32 |

**Table SVI. Comparison on main basal parameters between Chilean vs Mexican cohorts**

| **Parameter** | Group *L. reuteri*  (n=70)  Mexico Chile | | Group Placebo  (n=70)  Mexico Chile | | |
| --- | --- | --- | --- | --- | --- |
| Age (years) (x±s.d)  [min-max] | 39.4±9.97 | 38.8±11.01 | 40.2±11.1 | 40.4±15.05 | |
| Sex female (%) | 82 | 88 | 78 | 82 | |
| Tabaquism (%)  Duration (years) (x±s.d)  [min-max]  Frequency (cigarretes/day) (x±s.d)  [min-max] | 25*  5.8±9.0  3.0±2.7 | 30  5.3±8.0  3.2±2.1 | 8  3.9±6.0  2.6±3.4 | | 40  4.3±5.8  2.8±2.9 |
| Alcohol Ingestion (%)  Duration (years) (x±s.d)  [min-max]  Drinks/week (x±s.d)  [min-max] | 15  3.1±2.9 | 17  2.9±2.3 | 13  3.4±3.3 | | 12  3.1±3.0 |
| FODMAPS Ingestion  Low (%)  Moderate (%) | 85  15 | 88  12 | 80  20 | | 83  17 |
| Weight (Kg) (x±s.d)  [min-max] | 69.2±10.2 | 65.7±8.7 | 70.0±11.1 | | 68.2±10.1 |
| Height (mts) (x±s.d)  [min-max] | 1.60±0.07 | 1.58±0.05 | 1.61±0.08 | | 1.60±0.07 |
| Body Mass Index (Kg/m^2^) | 26.9±3.4 | 26.2±4.2 | 30.1±2.7 | | 29.2±3.8 |

*p<0.05, ** p<0.01, otherwise NS

**Table SVII. Comparison on main outcomes between Chilean vs Mexican cohorts**

| **Parameter (x±s.d.)** | **Group *L. reuteri***  **(n=68)**  **Mexico Chile** | | **Group Placebo**  **(n=67)**  **Mexico Chile** | | |
| --- | --- | --- | --- | --- | --- |
| Pre randomization GSRS-IBS (x±s.d) | 46.1±9.2 | 45.1±10.9 | 46.4±11.2 | 46.1±7.7 | |
| Pre randomization Bristol Scale (x±s.d) | 3.5±1.22 | 3.4±1.11 | 3.8±1.06 | 4.0±1.06 | |
| Pre randomization IBS QoL (x±s.d) | 101.8±15.46 | 104.0±21.41 | 103.2±15.70 | | 103.8±18.21 |
| Pre randomization anxiety (x±s.d) | 5.8±2.67 | 5.9±1.81 | 6.0±2.32 | | 6.3±2.62 |
| Pre randomization depression (x±s.d) | 3.5±2.02 | 3.1±1.95 | 3.4±2.26 | | 3.3±2.58 |
| Final Post Intervention GSRS-IBS (x±s.d) | 20.1±4.3* | 22.3±3.9* | 28.3±3.8 | | 30.3±4.3 |
| Final Post Intervention IBS QoL (x±s.d) | 134.5±11.81* | 131.9±14.3* | 106.1±6.19 | | 104.1±5.43 |
| Final Post Intervention anxiety (x±s.d) | 1.1±0.88* | 1.3±1.1* | 2.3±1.91 | | 2.5±2.94 |
| Final Post Intervention depression (x±s.d) | 0.8±0.61 | 1.1±0.89 | 1.2±1.32 | | 1.4±1.34 |

*p<0.05, ** p<0.01, otherwise NS

1. Wilcoxon signed rank test. 2-sided. [↑](#footnote-ref-1)
2. Wilcoxon signed rank test. 2-sided. [↑](#footnote-ref-2)
3. Wilcoxon rank sum test. 2-sided. [↑](#footnote-ref-3)
4. Wilcoxon signed rank test. 2-sided. [↑](#footnote-ref-4)
5. Wilcoxon signed rank test. 2-sided. [↑](#footnote-ref-5)
6. Wilcoxon rank sum test. 2-sided. [↑](#footnote-ref-6)
7. Wilcoxon signed rank test. 2-sided. [↑](#footnote-ref-7)
8. Wilcoxon signed rank test. 2-sided. [↑](#footnote-ref-8)
9. Wilcoxon rank sum test. 2-sided. [↑](#footnote-ref-9)
10. Wilcoxon signed rank test. 2-sided. [↑](#footnote-ref-10)
11. Wilcoxon signed rank test. 2-sided. [↑](#footnote-ref-11)
